# Identification of HM13 as a prognostic indicator and a predictive biomarker for immunotherapy in hepatocellular carcinoma

**DOI:** 10.1186/s12885-022-09987-2

**Published:** 2022-08-13

**Authors:** Genhao Zhang, Xianping Lv, Qiankun Yang, Hongchun Liu

**Affiliations:** 1 Department of Blood transfusion, Zhengzhou University First Affiliated Hospital, Zhengzhou, China; 2 Department of Medical Laboratory, Zhengzhou University First Affiliated Hospital, Zhengzhou, China

**Keywords:** HM13, Prognosis, Immune checkpoint inhibitors, HCC, Immunotherapy

## Abstract

**Background:**

Histocompatibility minor 13 (HM13) is a signal sequence stubbed intramembrane cleavage catalytic protein that is essential for cell signaling, intracellular communication, and cancer. However, the expression of HM13 and its prognostic value, association with tumor-infiltrating immune cells (TIICs) in the microenvironment, and potential to predict immunotherapeutic response in HCC are unknown.

**Methods:**

The HM13 expression, clinicopathology analysis, and its influence on survival were analyzed in multiple public databases and further verified in collected HCC and normal tissues by qRT-PCR and immunohistochemistry staining assay (IHC). Furthermore, the lentivirus vector encoding HM13-shRNA to manipulate HM13 expression was selected to investigate whether HM13 could influence the malignant growth and metastasis potential of HCC cells. Finally, significant impacts of HM13 on the HCC tumor microenvironment (TME) and reaction to immune checkpoint inhibitors were analyzed.

**Results:**

Upregulated HM13 was substantially correlated with poor prognosis in patients with HCC, and could facilitate the proliferation and migratory potential of HCC cells. Additionally, patients with high HM13 expression might be more sensitive to immunotherapy.

**Conclusions:**

HM13 might be a prognostic biomarker and potential molecular therapeutic target for HCC.

**Supplementary Information:**

The online version contains supplementary material available at 10.1186/s12885-022-09987-2.

## Background

Hepatocellular carcinoma (HCC), which accounts for almost 90% of all primary liver cancers, is one of the most frequent tumors and the leading cause of cancer-related deaths in the world [1]. The incidence and development of HBV or HCV infection, alcohol abuse, HIV co-infection, cirrhosis, and aflatoxin exposure may vary depending on the prevalence of risk factors such as HBV or HCV infection, alcohol abuse, HIV co-infection, cirrhosis, and aflatoxin exposure [2–4]. Furthermore, a growing body of evidence links diabetes, metabolic syndrome, and obesity to an increased risk of HCC in patients with non-alcoholic fatty liver disease (NAFLD) [5–8], implying that people who follow healthy lifestyle habits, such as a regular exercise routine and avoiding metabolic syndrome, may have a lower risk of developing HCC [9]. Most patients with early and intermediate stage HCC are known to receive therapeutic options such as liver resection, liver transplantation, radiofrequency ablation, and embolization [10]. However, due to a lack of biomarkers for early detection and the insidious nature of HCC, more than half of patients with HCC are already in an advanced stage by the time they are detected, at which point they are often unsuitable for curative therapy and the prognosis is still poor [11, 12]. Furthermore, many patients continue to experience tumor recurrence months or years later [13]. Although liver biopsy is the most reliable means of diagnosing HCC, it is an intrusive procedure with risks [14]. As a result, novel approaches to prognostic biomarkers and therapeutic targets are still needed to ameliorate dismal prognosis.

Histocompatibility small 13 (HM13) encodes a protein that localizes to the endoplasmic reticulum and catalyzes its intramembrane proteolysis following cleavage of some signal peptides from preproteins, which is required for the generation of lymphocyte surface (HLA-E) epitopes derived from MHC class I signal peptides [15, 16]. Growing evidence supports the association between HM13 expression, tumor-infiltrating immune cells (TIICs) [16, 17], and cancer [18, 19]. However, the function of HM13 in HCC has never been reported before. This study aimed to investigate the relationship between HM13 expression profiles, clinicopathological features, immune infiltration, and immunotherapy responses in HCC patients and to explore the role of HM13 in the development of HCC, which may contribute to our understanding of the mechanisms of HCC.

## Methods

### Bioinformatics analysis by public databases

A pan-cancer study of HM13 expression was performed using the Gene Expression Profiling Interactive Analysis (GEPIA) database [20]. The GEO, TCGA, and ICGC databases were used to investigate the relationship between HM13 expression and clinicopathologic features in samples with and without HCC. The human protein atlas database (HPA) was used to compare the protein expression of HM13 in normal and HCC tissues (www.proteinatlas.org). The researchers utilized R software to do a Cox regression analysis to see if HM13 was an independent predictive factor in the TCGA study. The potential of HM13 in predicting immunotherapeutic response was investigated using the IMvigor210 cohort.

### Clinical data

Fifty-nine HCC samples and 50 normal tissues were selected from patients who underwent liver biopsy. Before surgery, none of the patients had had radiation or chemotherapy, and those with missing data were eliminated. Two prominent pathologists evaluated and co-diagnosed all pathological data.

### Quantitative real-time PCR, immunohistochemistry staining, and western-blotting assay

As previously described [21], we used quantitative real-time PCR (qRT-PCR, F603101, Sangon Biotech, China) and immunohistochemical staining test (IHC) to compare the mRNA and protein expression levels of the HM13 and CHOP genes in HCC and normal tissues. The following are the primer sequences: HM13, Forward: GATCCGCATAACGGCAGTG, Reverse GAGCCCCAAGAGTGTGCAG, CHOP, Forward: GGAAACAGAGTGGTCATTCCC, Reverse CTGCTTGAGCCGTTCATTCTC, and β-ACTIN, Forward: CGTGGGCCGCCCTAGGCACCA, Reverse TTGGCTTAGGGTTCAGGGGGG. The sections were subjected to IHC after being heated in citrate buffer for antigen retrieval, quenching endogenous peroxidase with 2% hydrogen peroxide, and blocking with 5% bovine serum albumin (BSA, A602449, Sangon Biotech, China). After that, the slices were incubated at 4 °C for the duration of the night with an anti-HM13 (ab247061, Abcam) antibody and an isotype control. DAB chromogen (A690009, Sangon Biotech, China) was used to stain the sections progressively after washing with PBST (Phosphate Buffer Solution with Tween-20). Two competent pathologists performed the IHC findings assessment using the single-blind and unified criteria procedures. To distinguish between low/loss (4) and high (> 4) expression of HM13 in HCC and normal tissues, a final score of the sum of the extent of expression score (no positive cells = 0, 10% = 1, 10–50% = 2, positive staining of > 50% = 3) and intensity score (negative = 0, weak = 1, moderate = 2, strong = 3) was used. Finally, the western-blotting (WB) assay was used to examine the knockdown effect of HM13 protein in MHCC97L cells. RIPA buffer (20–188, Sigma) was applied to the whole-cell lysates of MHCC97L cells. A 12% SDS-PAGE gel was used to separate 30 g of protein, which was then transferred to a PVDF membrane. Membranes were incubated with anti-HM13 (1:1000, ab247061, Abcam) and anti-β-ACTIN (1:2000, ab8227, Abcam) antibodies at 4 °C overnight. Blocking was performed with a 5% blocking buffer. Following that, membranes were treated for 1 hour with the secondary antibody Anti-HRP Rabbit (1:10000, 207674, Abcam). To produce protein bands, an ECL reagent (P0018S, Beyotime, China) was utilized. Using Image Lab software, the relative expression of HM13 was normalized to β-ACTIN. The experiments covered in our study were all set up with 2 additional independent controls.

### Cell lines and cell culture

The Shanghai Institute of Cells (Chinese Academy of Science, Shanghai, China) provided cell lines (HepG2, Hep3B, HuH7, and MHCC97L). Cells were grown in prescribed DMEM medium (E600008, Sangon Biotech, China) with 10% fetal bovine serum (FBS, E600001, Sangon Biotech, China) at 37 °C with 5% CO_2_ in 100% humidity.

### Plasmids construction and cell infection

The Lentiviral vector encoding HM13’s Lenti-shRNA vector system (PCDH-GFP) (HM13-shRNA sequence: CCGGGTGCCTGAAACAATCACCAGCCACTCGAGTGGCTGGTGATTGTTTCAGGCACTTTTT) were chosen. Control vectors were shRNA controls with a non-targeting sequence. The HM13-shRNA was selected for creating stable knockdown (KD) cell lines using lipofectamine™ 3000 transfection reagent (L3000001, Invitrogen, USA) according to the manufacturer’s instructions, and MHCC97L cells were grown in DMEM (E600008, Sangon Biotech, China) supplemented with 10% FBS (E600001, Sangon Biotech, China) for cell infection.

### Cell proliferation assay

The cell counting kit-8 assay was used to determine the vitality of the cells (CCK-8, E606335, Sangon Biotech, China). Cells were seeded at a density of 1 × 10^5^ cells per well in 96-well cell culture clusters and cultivated for 1 day, 2 days, and 3 days, respectively. After culture, 10 μL CCK-8 solution was applied to each well, and the absorbance was measured with a microplate reader at 450 nm within 4 hours.

### Wound-healing assay

In a serum-free medium, cells were grown to about 80% confluence for 12 hours before being scratched with a sterile pipette tip. After scratching, cells were grown for the following 24 hours. The migrative ability was quantified and analyzed by measuring the scratch area which was not covered by cells at 100 x magnification. Three independent duplicates were needed to ensure the accuracy of this assay and the wound closure rate was calculated as [1 - (wound area / original wound area)] from photographs acquired at 24 h.

### Transwell assay

The 24-Transwell chambers were used in the invasion experiment (3244, Corning Inc., Corning, USA). After coating transwell filters with Matrigel (BD Biosciences, USA), cultured cells were resuspended at a density of 1 × 10^4^ cells per mL in 200 μL serum-free DMEM (E600008, Sangon Biotech, China), applied to the Matrigel membrane, and plated in the upper chamber while the bottom chamber was filled with 500 μL DMEM supplemented with 10% FBS (E600001, Sangon Biotech, China). Cells adhering to the bottom chamber were stained with 0.1% crystal violet in PBS for 15 minutes and counted under microscopy at 200 x magnification after being cultured at 37 °C for 48 hours.

### Immune infiltrates analysis and signaling pathway analysis

To evaluate the potential relationships between HM13 expression profiles and TIICs in the HCC microenvironment, the TCGA database was used to gauge the proportions of TIICs via CIBERSORT [22] and the Timer database [23]. Subsequently, to explore the signaling pathways involved in HM13 mediated effects, Gene set enrichment analysis (GSEA) was performed to distinguish the altogether cautioned Hallmark items with FDR < 0.05.

### Genetic alterations and TMB analysis

The somatic variants data of 350 HCC patients were downloaded from TCGA to analyze the difference of genetic alterations between the high- and low-HM13 expression subgroups with R package “maftools”, and the tumor mutation burden (TMB) of each patient was subsequently assessed.

### HM13 methylation level analysis

The data on HM13 copy number variation (CNV) and methylation level was obtained from the cBioPortal web platform (https://www.cbioportal.org/), and a comparison of the varying HM13 gene expressions in HM13 copy number variation groups and the correlation between HM13 methylation level and HM13 gene expression were performed. TCGA data was utilized to determine changes in the promoter methylation level of HM13 between HCC and normal tissues using the UALCAN web tool (http://ualcan.path.uab.edu/). The MethSurv online tool (https://biit.cs.ut.ee/methsurv/) was utilized to investigate the HM13 methylation level’s predictive value in the TCGA-LIHC cohort.

### Statistical analysis

**Fig. 1:**
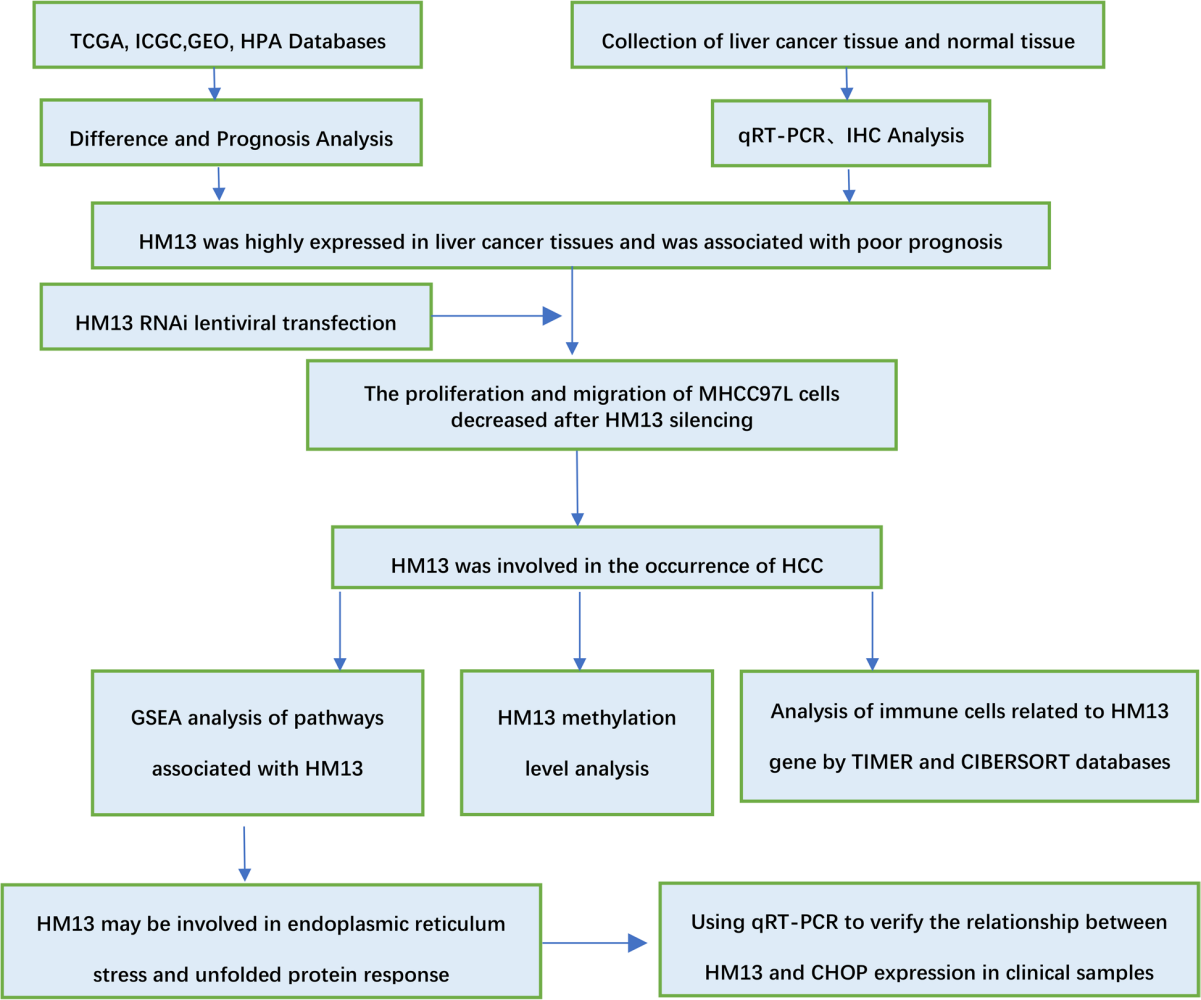
A flow chart

R software was used to conduct statistical analysis (Version 4.0.2). When applicable, categorical data were compared using the Pearson chi-square test or Fisher exact test, and quantitative data between the two groups were compared using the t-test. The influence of HM13 expression on the OS rate of patients with HCC was assessed using Kaplan-Meier survival analysis with the log-rank test. The link between HM13 expression and overall survival, as well as other clinical variables, was investigated using a Cox proportional hazards regression model. One-way analysis of variance (ANOVA) was performed to analyze several groups. For HM13 expression, a *P* value of 0.05 was considered statistically significant. A flow chart was shown in Fig. 1.

## Results

### Pan-cancer analysis of HM13 expression in TCGA database

**Fig. 2:**
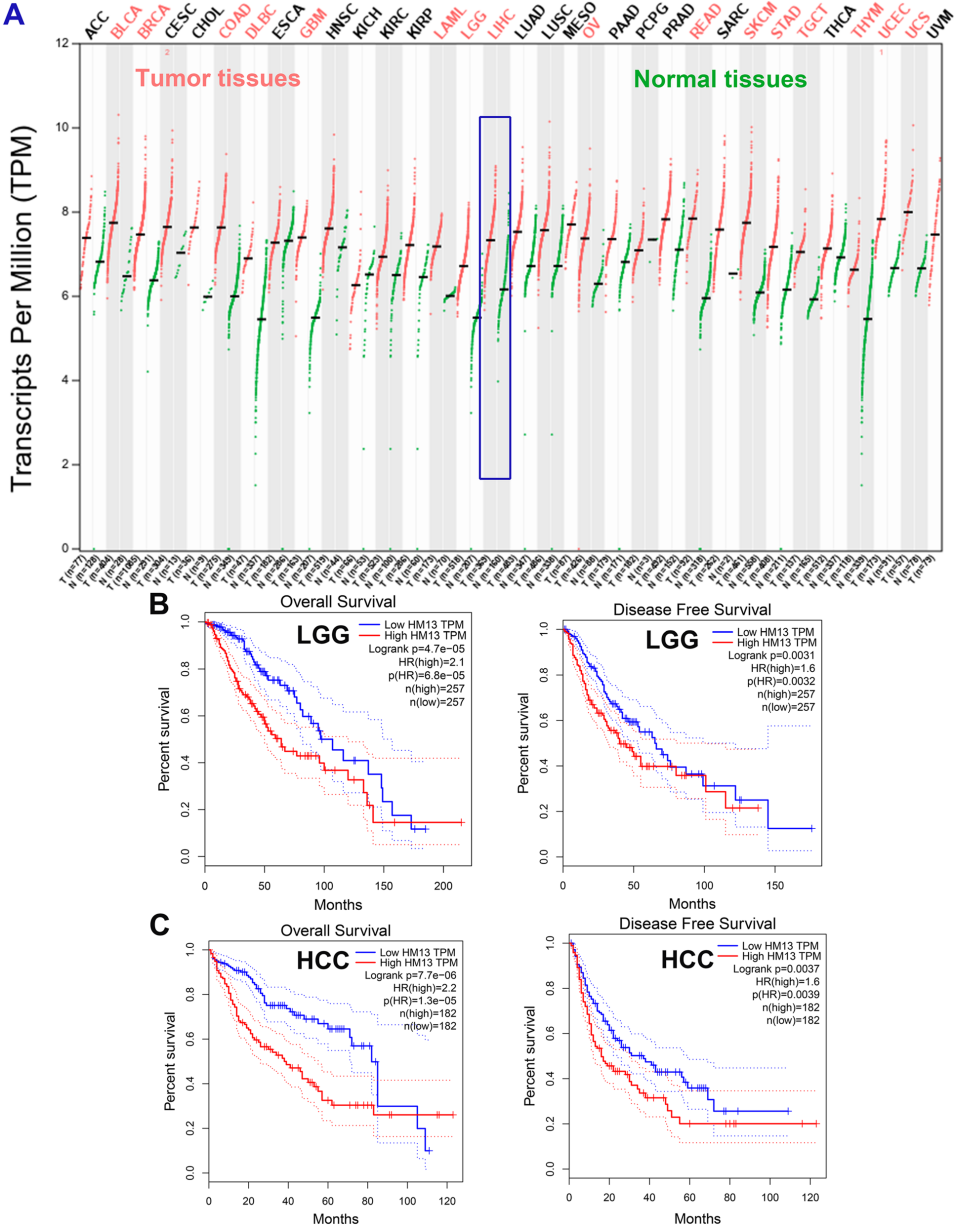
Pan-cancer analysis of HM13 expression in TCGA. **A** HM13 expression in 33 types of cancers. High HM13 expression was related to overall survival and disease-free survival in patients with LGG (**B**) and LIHC (**C**)

The results of the pan-cancer analysis performed in GEPIA showed that HM13 was significantly increased in various tumor tissues, including BLCA, BRCA, COAD, DLBC, GBM, KAML, LGG, LIHC, OV, READ, SKCM, STAD, TGCT, THYM, UCEC, and UCS, when compared to normal tissues (Fig. 2A, *p* < 0.01), however, HM13 was exclusively linked to poor overall survival and disease-free survival in patients with LGG (Fig. 2B, *p* < 0.05) and LIHC (Fig. 2C, *p* < 0.05). Considering that clinical samples of LGG were difficult to collect, and we had collected clinical samples of HCC and normal tissues, subsequently, we focused on the role of HM13 in HCC.

### HM13 expression is significantly increased in HCC

**Fig. 3:**
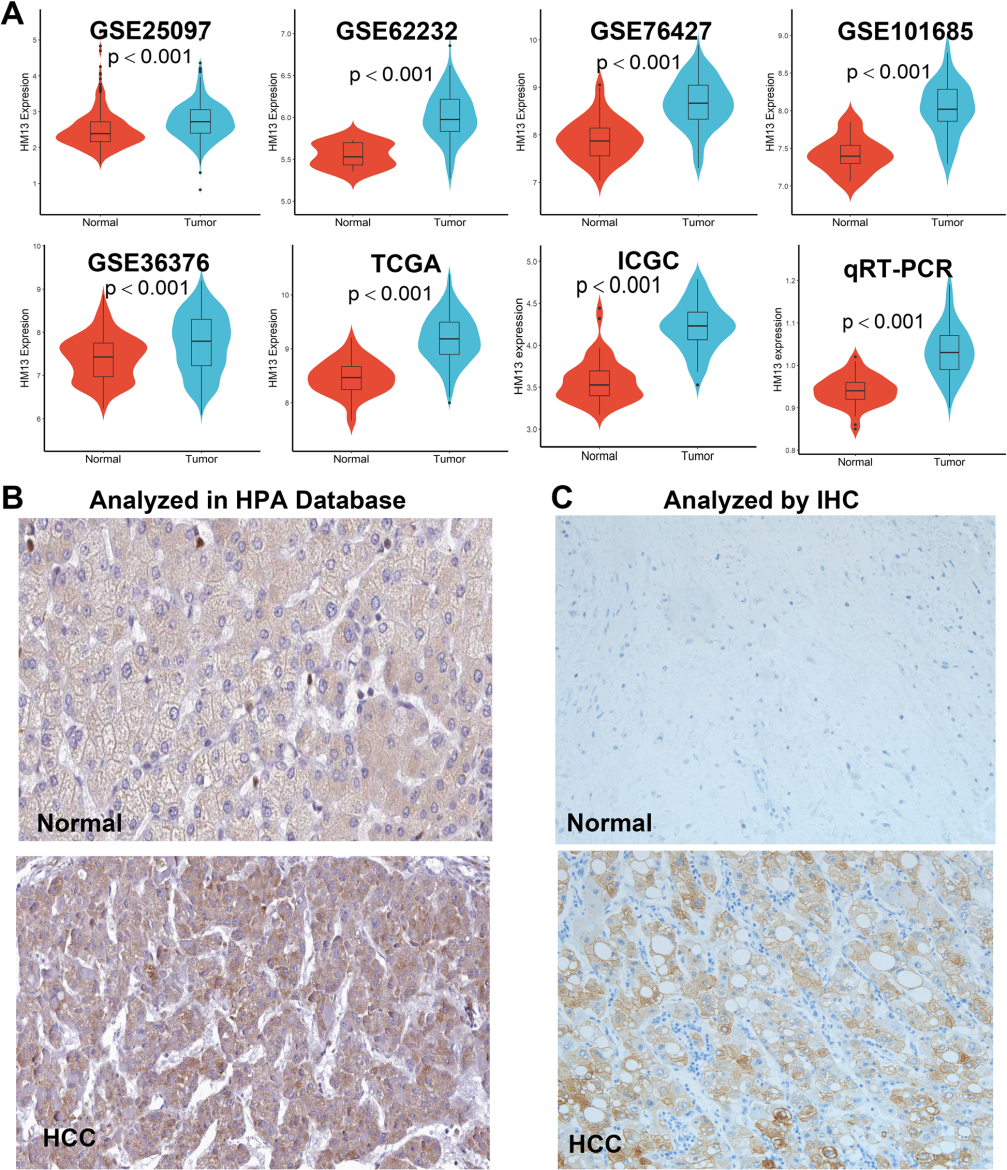
HM13 was overexpressed in HCC. **A** HM13 mRNA expression was analyzed in multiple databases. **B** HM13 protein expression was analyzed in HPA databases. **C** HM13 protein expression was analyzed by IHC

The results analyzed in multiple databases including GSE25097, GSE62232, GSE76427, GSE101685, GSE36376, TCGA, ICGC, and collected clinical samples showed that HM13 expression level in HCC tissues was significantly higher than that in normal liver tissues (Fig. 3A, *p* < 0.05), and differential protein expression of HM13 between normal and HCC tissues analyzed by HPA (Fig. 3B) and IHC (Fig. 3C) were consistent with the above results. Among these clinical samples, we detected 3 cases of HM13^High^ and 47 cases of HM13^Low/loss^ in normal tissues, 26 cases of HM13^High^ and 33 cases of HM13^Low/loss^ in tumor tissues (*p* < 0.05), respectively. In summary, HM13 expression levels, both mRNA and protein expression, were significantly increased in HCC.

### HM13 expression increased with the progression of HCC

**Fig. 4:**
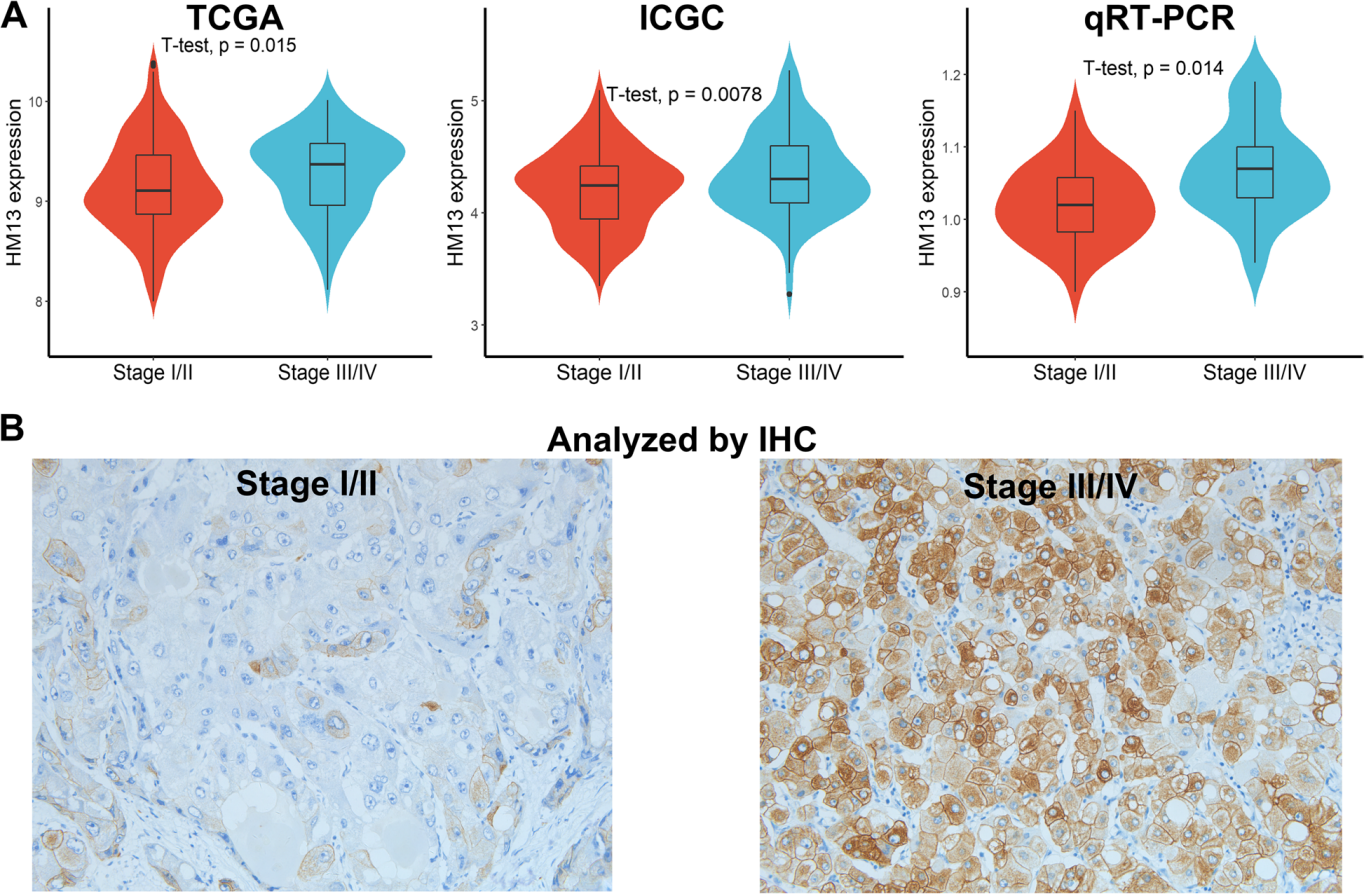
HM13 expression increased with the progression of HCC. **A** HM13 mRNA expression in early and later stages of HCC. **B** HM13 protein expression in early and later stages of HCC

The relationship between HM13 expression and tumor progression was explored and the results revealed that both HM13 mRNA (Fig. 4A, *p* < 0.05) and protein expression (Fig. 4B, *p* < 0.05) significantly increased in later stages of HCC compared with that in early stages. Among the 59 tumor tissues, we detected 12 cases of HM13^High^ and 30 cases of HM13^Low/loss^ in early stages tissues, and 14 cases of HM13^High^ and 3 cases of HM13^Low/loss^ in later stages tissues (*p* < 0.05), respectively.

Moreover, HM13 expression was also related to grade and relapse in HCC patients (Table 1).

**Table 1:** Relationship between HM13 expression and clinical pathological features

Clinicopathological parameter	Case(n)	HM13 expression		χ2	*p* Value
		High	Low/loss		
Age (years)					
≤ 60	27	12	15	0.002	0.957
> 60	32	14	18		
Gender					
Male	45	20	25	0.010	0.916
Female	14	6	8		
HBV Infection					
Yes	41	19	22	0.281	0.595
No	18	7	11		
Recurrence					
Yes	29	17	12	4.901	0.0268
No	30	9	21		
TNM Stage					
I &II	42	12	30	14.201	0.0002
III&IV	17	14	3		
Grade					
G1 & G2	35	9	26	11.761	0.0006
G3 & G4	24	17	7		

### Increased HM13 expression indicated a poor survival outcome of HCC

**Fig. 5:**
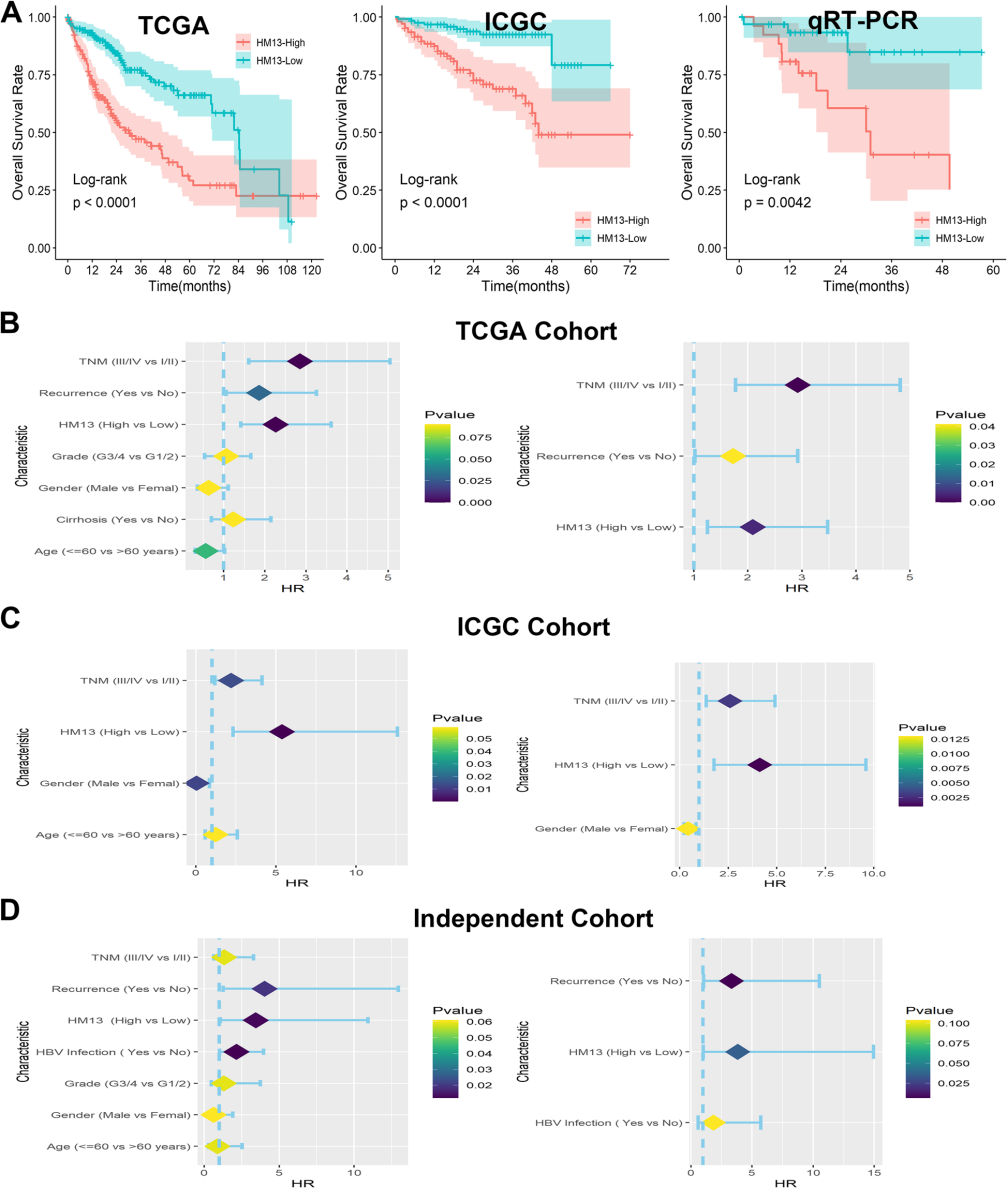
Overexpressed HM13 indicated a poor prognosis for HCC. **A** Kaplan–Meier plots of HM13 expression in three HCC datasets. **B**-**D** Univariate and multivariate Cox regression analysis revealed that HM13 expression was an independent prognostic factor in three HCC databases

Kaplan-Meier analysis was performed to explore the relationship between HM13 expression and overall survival outcome and the results revealed that patients with lower HM13 expression had a better overall survival rate (Fig. 5A, *p* < 0.05). Furthermore, we found that HM13 expression was an independent prognostic factor for HCC patients by Cox proportional regression model in TCGA (Fig. 5B, *p* < 0.05), ICGC (Fig. 5C, *p* < 0.05), and clinical sample cohort (Fig. 5D, *p* < 0.05).

### HM13 expression was involved in immune infiltration in HCC

**Fig. 6:**
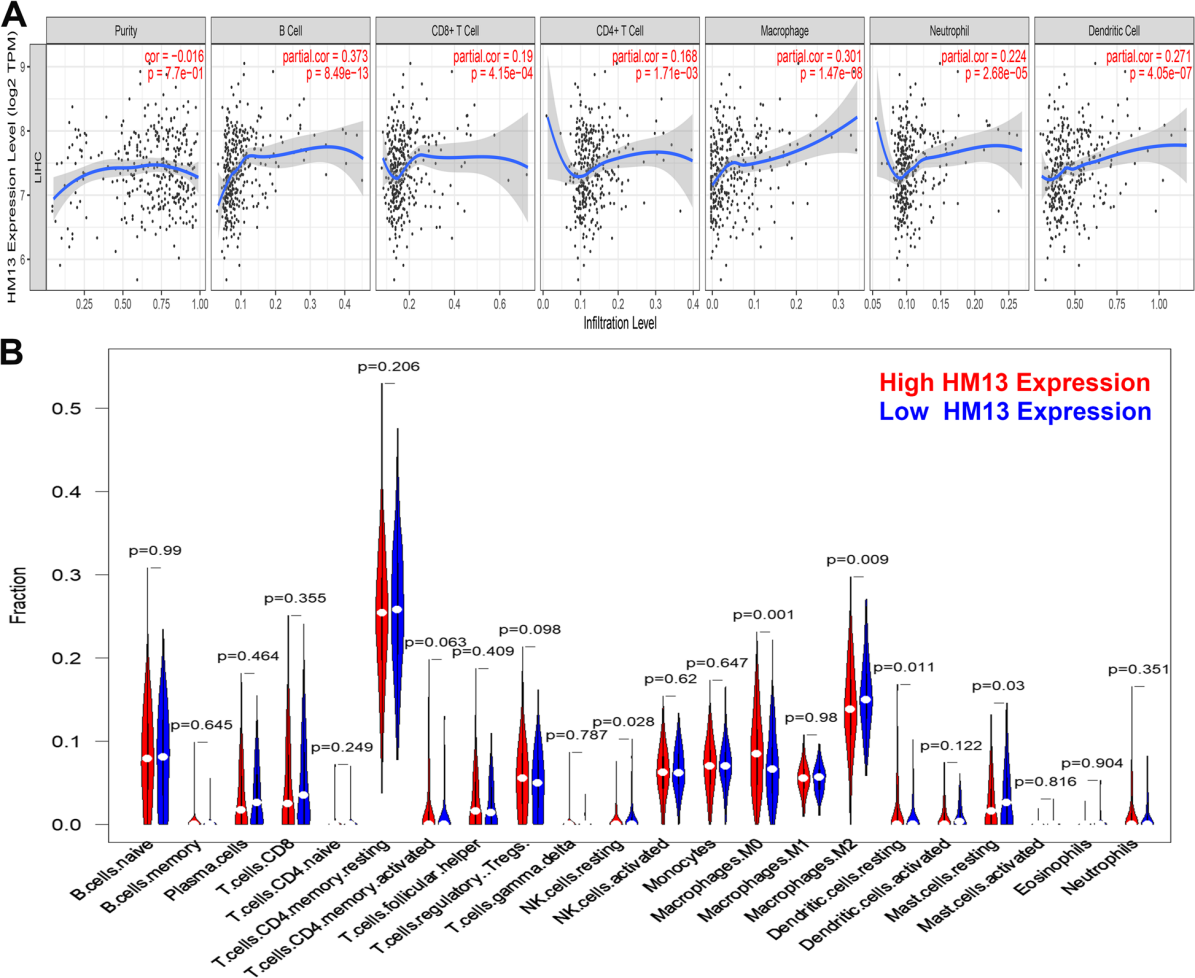
HM13 expression was involved in immune infiltration in HCC. TIICs analysis by Timer database (**A**) and CIBERSORT (**B**)

The TIMER database was used to assess the correlation between HM13 expression and TIICs levels using the Pearson method and the result showed that HM13 was significantly related to infiltration levels of B cell, CD4+ T cell, CD8+ T cell, macrophage, neutrophil, and dendritic cell (Fig. 6A). Furthermore, difference analysis of TIICs infiltration levels between high- and low-HM13 expression subgroups was assessed by Wilcoxon signed-rank test based on the CIBERSORT algorithm and the result demonstrated that HCC patients with higher HM13 expression had modestly increased ratios of macrophages M0 and resting dendritic cells, while patients with lower HM13 expression had significantly elevated ratios of resting NK cell, macrophages. M2 and resting mast cells (Fig. 6B).

### Relationship between HM13 and immune checkpoint genes and the potential of HM13 in predicting immunotherapeutic response in HCC

**Fig. 7:**
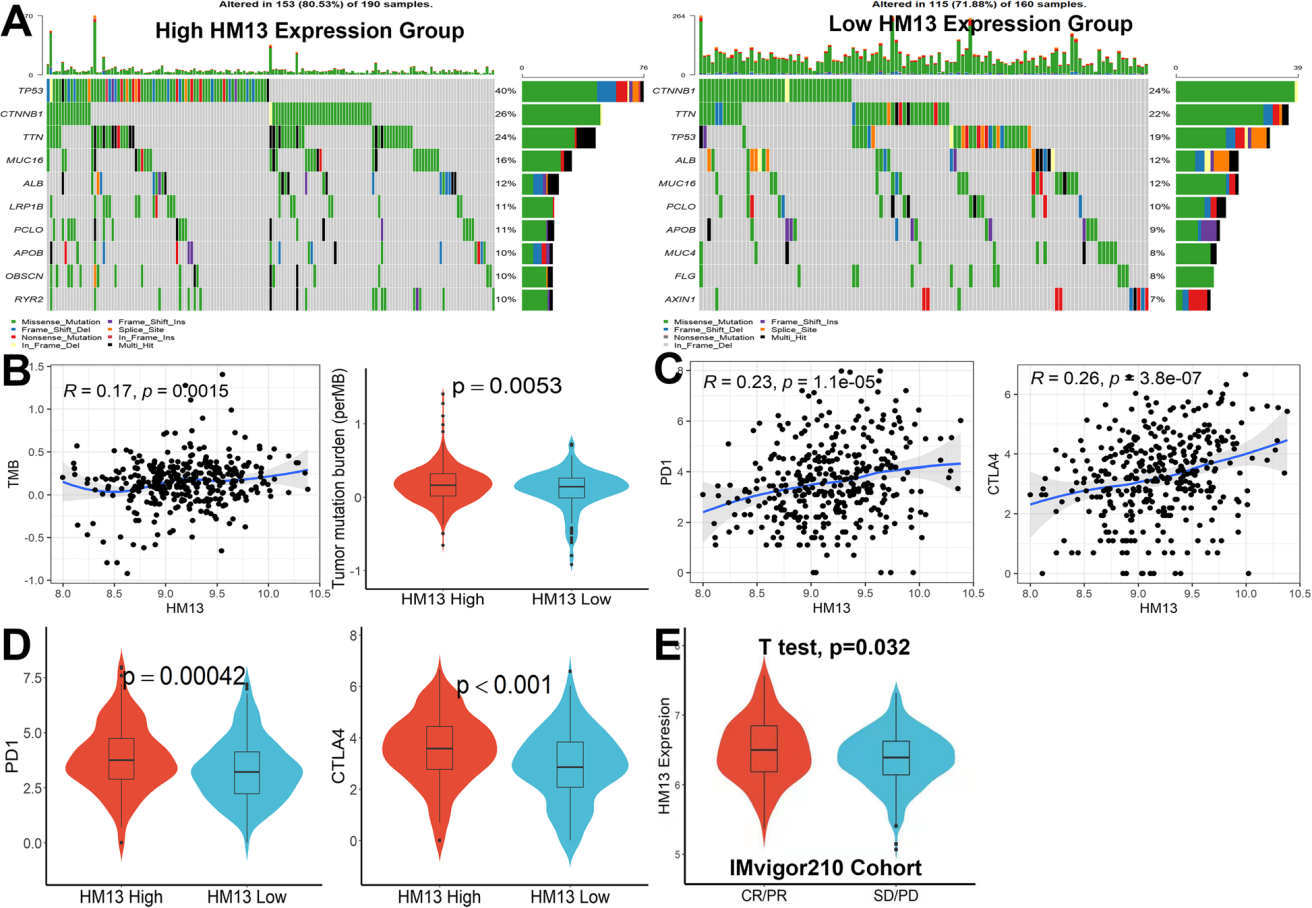
Relationship between HM13 and immune checkpoint genes and the potential of HM13 in predicting immunotherapeutic response in HCC. **A** Oncoplots of top 10 mutated genes in high- and low-HM13 expression subgroups. **B** Correlation and difference analysis of HM13 expression and TMB. **C**-**D** Correlation and difference analysis of HM13 and PD1 and CTLA4 expression. **E** HM13 expression in different anti-PD1 and antiCTLA4 responses of patients from the IMvigor210 cohort

The results of genetic alterations analysis indicated that the mutation rates of the top 10 most significantly mutated genes were remarkably different in the two subgroups (Fig. 7A). The most significantly mutated gene was TP53 in the HM13-high subgroup, while CTNNB1 was the most significantly mutated gene in the HM13-low subgroup. Subsequently, the TMB of each patient was assessed. HM13 expression was closely related to TMB, and patients with higher HM13 expression had modestly increased ratios of TMB (Fig. 7B, *p* < 0.05). In the following, we found the expression of PD1 and CTLA4 were significantly related to the expression of HM13 (Fig. 7C, *p* < 0.001), and patients with higher HM13 expression had significantly increased PD1 and CTLA4 (Fig. 7D, *p* < 0.001), indicating that immune checkpoint inhibitors (ICIs) treatment was more effective for patients with higher HM13 expression. Finally, the IMvigor210 cohort was used to analyze the potential of HM13 in predicting immunotherapeutic response and we found that patients with high HM13 expression might be more sensitive to immunotherapy (Fig. 7E, *p* < 0.05).

### HM13 promoted the proliferation and migratory potential of HCC cells

**Fig. 8:**
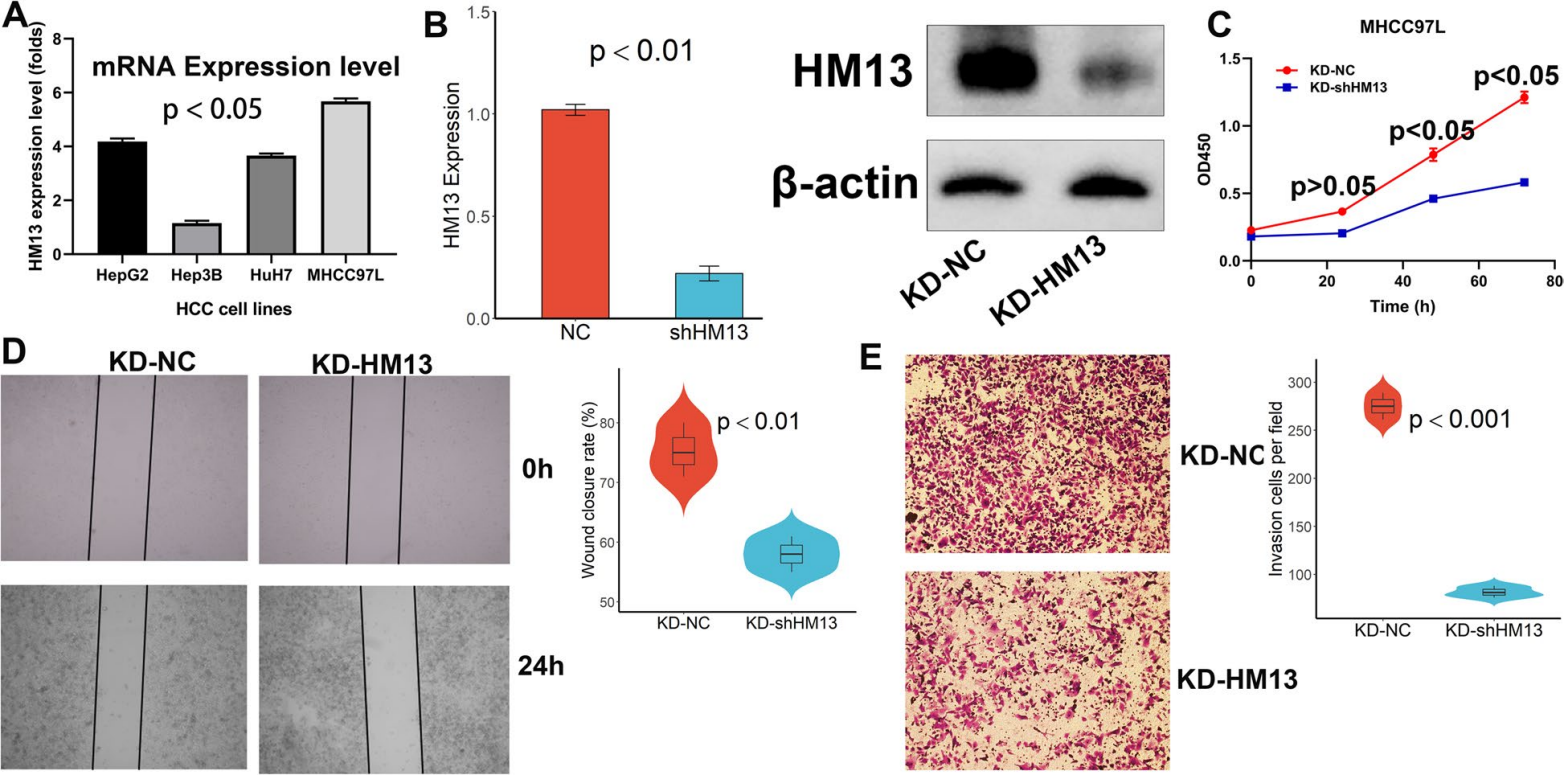
HM13 promoted the proliferation and migratory potential of HCC cells. **A** HM13 expression in four HCC cell lines. **B** Stable cell lines for knockdown of HM13. **C** CCK-8 assay. **D** Wound-healing assay. **E** Transwell assay

The HM13 expression level was assessed in four HCC cell lines including HepG2, Hep3B, HuH7, and MHCC97L (Fig. 8A, *p* < 0.05). Next, we selected the lentivirus vector encoding HM13-shRNA to manipulate HM13 expression in MHCC97L cell lines. HM13 expressions were confirmed by qRT-PCR (Fig. 8B, *p* < 0.001). To investigate the influence of HM13 on the malignant growth and metastasis potential, cell proliferation assay, wound-healing assay, and transwell assay were performed. For cell proliferation ability, the results of CCK-8 showed that the proliferation rate of down-regulated HM13 was decreased (Fig. 8C, *p* < 0.05). For the wound-healing assay, we found that HM13 knockdown remarkably inhibited wound closure (Fig. 8D, *p* < 0.01). Meanwhile, in transwell chambers with Matrigel assay, HM13 knockdown dramatically reduced cell invasion (Fig. 8E, *p* < 0.001).

### Signaling pathway analysis of HM13-mediated effects

**Fig. 9:**
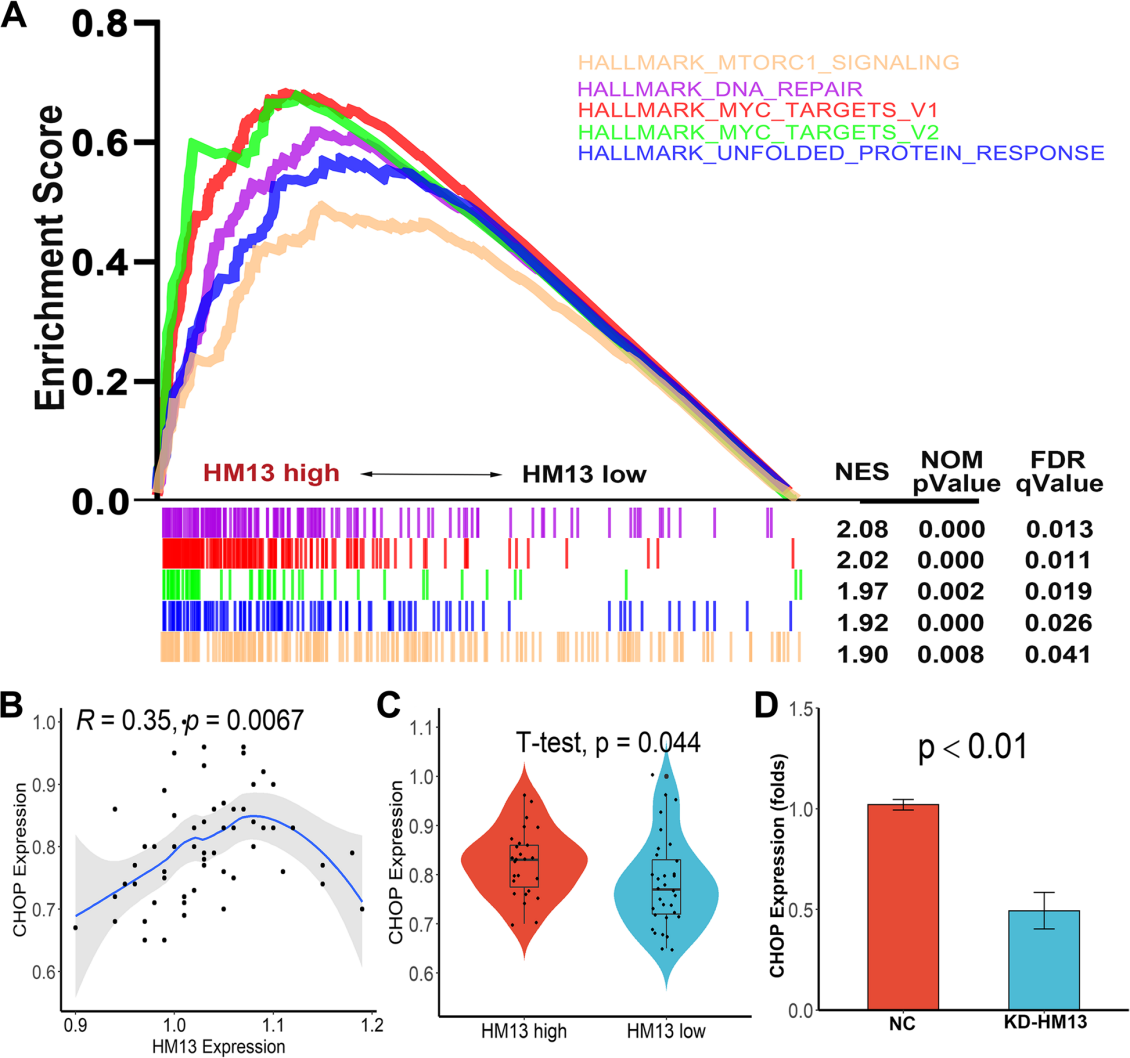
Signaling pathway analysis of HM13-mediated effects **A** GSEA analysis. **B** Correlation analysis. **C** Difference analysis. **D** CHOP expression was significantly reduced after HM13 knockdown

According to the results of the GSEA, the expression of HM13 was significantly correlated with HALLMARK DNA REPAIR and HALLMARK UNFOLDED PROTEIN RESPONSE (Fig. 9A), suggesting that HM13 may be associated with endoplasmic reticulum stress and unresponsive protein response (UPR). Excitingly, consistent with the above analysis, we used qRT-PCR detection to find that HM13 was significantly correlated with the expression of URP-related pathway molecule CHOP (Fig. 9B, *p* < 0.001), and CHOP was also highly expressed in HCC tissue samples with high HM13 expression (Fig. 9C, *p* < 0.05). In addition, the expression of CHOP was also significantly reduced after HM13 knockdown (Fig. 9D, *p* < 0.01). All these suggest that HM13 may play an important regulatory role in the activation of PERK/CHOP pathway.

### HM13 Hypomethylation in HCC

**Fig. 10:**
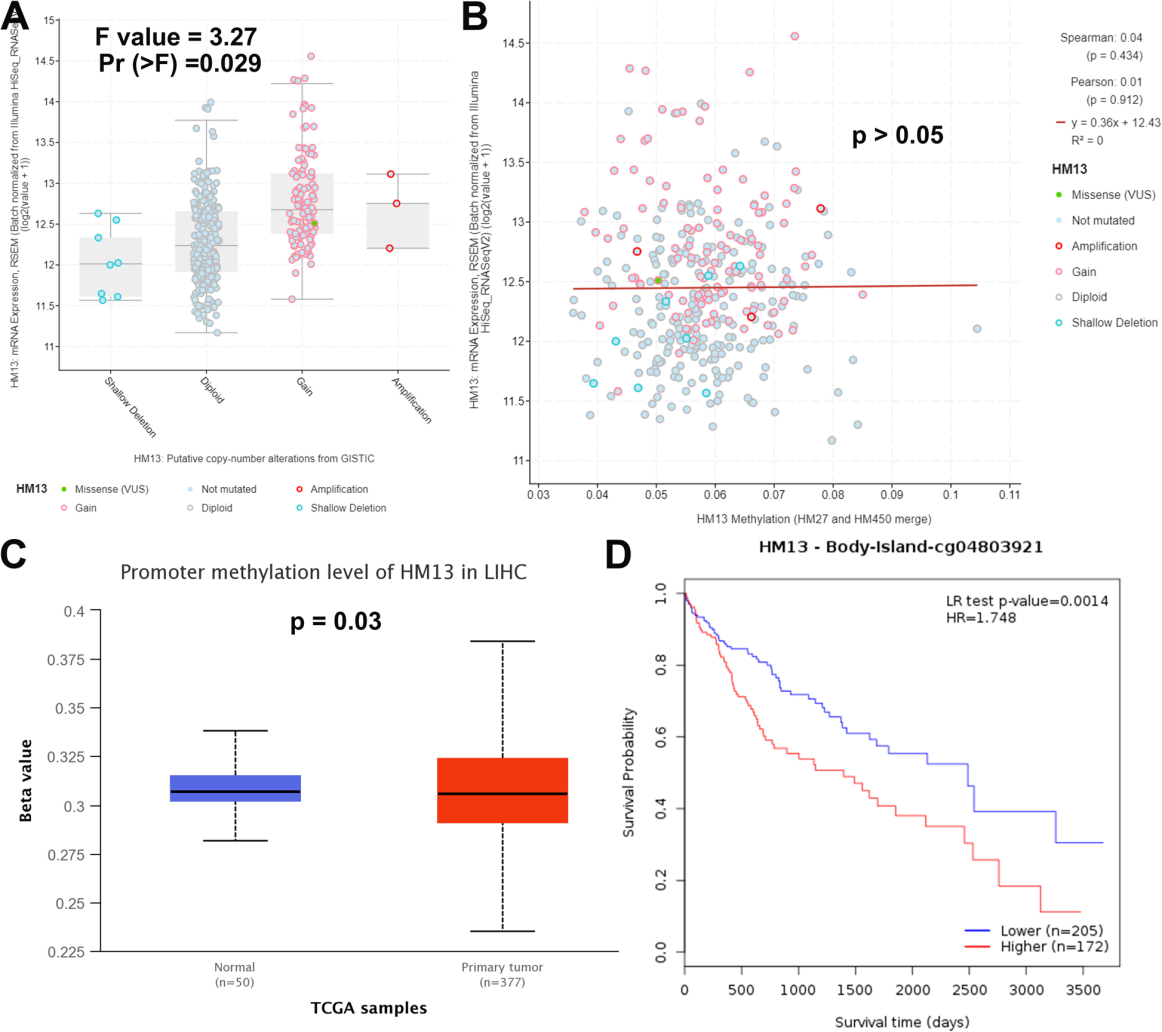
HM13 Hypomethylation in HCC. **A** Expression levels in different CNV of HM13. **B** Correlation analysis between HM13 methylation and its expression. **C** The promoter methylation of HM13 in HCC. **D** Kaplan-Meier survival of the promoter methylation of HM13

Patients with HM13 CNV gain had a greater level of HM13 expression (Fig. 10A, *p* < 0.05), according to the cBioPortal, however HM13 methylation was not connected to HM13 gene expression (Fig. 10B, *p* > 0.05). The promoter methylation of HM13 in HCC tissues was substantially greater than in surrounding normal tissues (Fig. 10C, *p* < 0.05), according to the UALCAN. Patients with decreased HM13 methylation had a better overall survival (Fig. 10D, *p* < 0.01), according to the MethSurv online tool.

## Discussion

With the characteristics of high malignancy and rapid progress, more than half of patients with HCC were diagnosed at a late stage with a worse clinical outcome and would die within 3–6 months after diagnosis, which mainly resulted from tumor location, tumor cell invasion, and metastasis, tumor recurrence, and body characteristics, although significant advances have been achieved in the medical fields of anticancer therapeutics [1, 24–26]. Various genetic variations have a role in the start and progression of HCC. Identifying genes that are dysregulated during tumor growth can therefore aid in improving prognosis and therapy options. HM13, an intramembrane cleavage catalytic protein of signal sequence stubs, was proved to play an important role in the maturation of tail-anchored protein [16] and dislocation of TGF-β1from the endoplasmic reticulum [15] and be necessary for the removal of the signal peptide that remains attached to the hepatitis C virus core protein after the initial proteolytic processing of the polyprotein [27]. Meanwhile, HM13 was identified to increase the levels of cytokines in EGFRvIII secretion profiles and facilitate tumor progression and development of Glioblastoma in vivo and vitro while EGFRvIII was the most common mutation [18, 28, 29]. Interestingly, the focus of our research was on elucidating the mechanism by which down-regulated HM13 might impact the malignancy of HCC cells.

As can be observed from our findings, HM13 was considerably elevated and connected to poor prognosis and survival rate in patients with HCC, regardless of whether the data came from public databases or clinical samples obtained, which was consistent with the findings in GBM patients [18]. It is worth noting that in normal tissues, we only detected 3 cases of high expression of HM13, and the rest were extremely low, and even no expression of HM13 was detected. To better highlight the high expression level of HM13 in HCC, we selected normal tissues that do not express HM13 for display. In addition, we reconfirmed our results with public databases including Kaplan-Meier Plotter and the Human Protein Atlas database. We found that patients with increased HM13 expression had poor overall survival, whether by Kaplan-Meier Plotter or Kaplan-Meier Plotter (Supplementary Fig. S1). Subsequently, we explored the role of HM13 methylation in HCC. According to the cBioPortal, we found that although HM13 methylation was not associated with HM13 gene expression, patients with increased HM13 CNV had higher levels of HM13 expression. According to the UALCAN, the promoter methylation of HM13 was significantly higher in HCC tissues than in surrounding normal tissues. According to the MethSurv online tool, patients with reduced HM13 methylation had better overall survival. These indicate that HM13 methylation is also involved in the occurrence and progression of HCC, which deserves more in-depth research in future work. Finally, we aimed to investigate whether HM13 could influence the malignant growth and metastasis potential of HCC cells after selecting the lentivirus vector encoding HM13-shRNA to manipulate HM13 expression. The results obtained indicated that down-regulated HM13 could inhibit the proliferative and metastatic potential of HCC cells, further supporting the notion of the enhancement role played by HM13 in HCC.

Importantly, our study expounded on the relationship between HM13 expression and TME in HCC. TIICs have been reported to play a key regulatory role in tumorigenesis and the progression of HCC [30–32]. We found a significant difference in the proportion of some TIICs between the HM13-high and HM13-low subgroups in TCGA. Furthermore, we found that the TMB, expression of PD1 and CTLA4 were significantly and positively correlated with the expression of HM13, and their levels were significantly higher in the HM13-high subgroup than in the HM13-low subgroup. We also found that patients with high HM13 expression might be more sensitive to immunotherapy in the IMvigor210 cohort. The above data suggest that HM13 expression can accurately predict the immune profile of HCC, and patients with high HM13 expression may be more responsive to ICI therapy.

Last but not least, there are certain limits to our research. First, after screening public databases, the expression levels and survival prediction values of HM13 were validated in a clinical sample cohort; however, the sample size was small, and additional verification in randomized controlled trials was required. Furthermore, in public databases, the expression of PD1 and CTLA4 was utilized to predict patient response to ICI treatment; thus, HCC patients treated with ICIs were required to evaluate the true predictive usefulness of HM13.

## Conclusion

In conclusion, our study revealed for the first time that upregulation of HM13 was significantly associated with poor survival outcomes in HCC patients and promoted cell proliferation and migration capacity. In addition, HM13 may play an important role in the regulation of TIICs and immune checkpoint gene expression in the immune microenvironment of HCC. HM13 has the potential to be a predictive biomarker as well as a molecular therapeutic target for HCC.

## Supplementary Information

**Additional file 1.**

**Additional file 2: Fig. S1.** Increased HM13 expression had poor overall survival, whether in Kaplan-Meier Plotter (A) or HPA (B) database.

## Data Availability

The datasets used and/or analyzed during the current study are available from the corresponding author on reasonable request.

## Abbreviations

HM13: Histocompatibility minor 13
TIICs: Tumor-infiltrating immune cells
IHC: Immunohistochemistry staining assay
TME: Tumor microenvironment
HCC: Hepatocellular carcinoma
NAFLD: Non-alcoholic fatty liver disease
GEPIA: Gene Expression Profiling Interactive Analysis
HPA: Human protein atlas
TMB: Tumor mutation burden
ICIs: Immune checkpoint inhibitors
FBS: Fetal bovine serum
CCK-8: Cell counting kit-8 assay
CNV: Copy number variation
