# Supplementary material for: Identification of HM13 as a prognostic indicator and a predictive biomarker for immunotherapy in hepatocellular carcinoma
Source: BMC Cancer. 2022 Aug 13;22:888. doi: 10.1186/s12885-022-09987-2 (PMC9375928; doi:10.1186/s12885-022-09987-2)

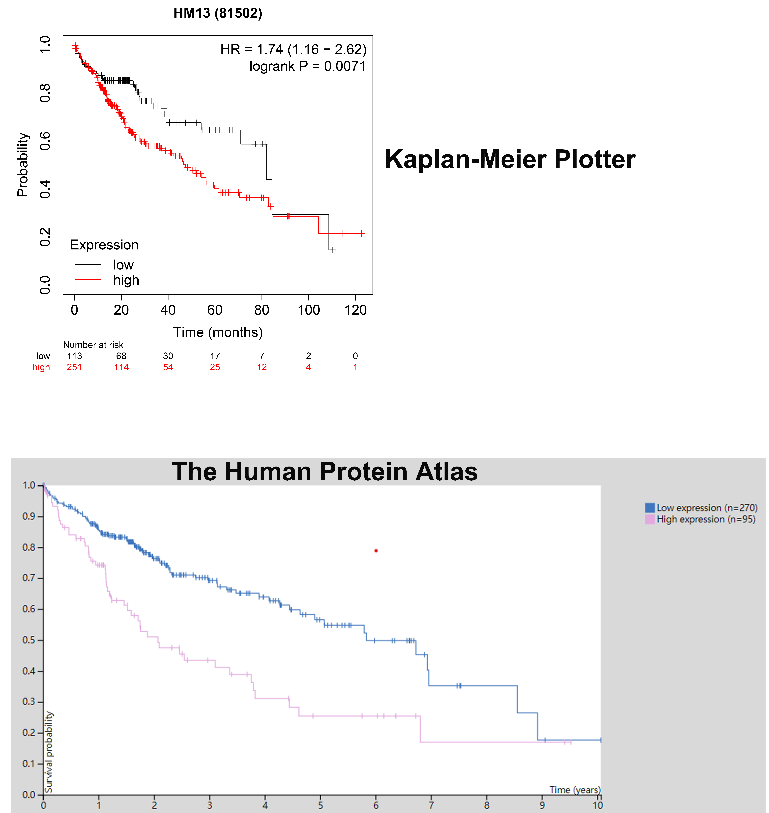


Figure S1 Increased HM13 expression had poor overall survival, whether in Kaplan-Meier Plotter (A) or HPA (B) database.


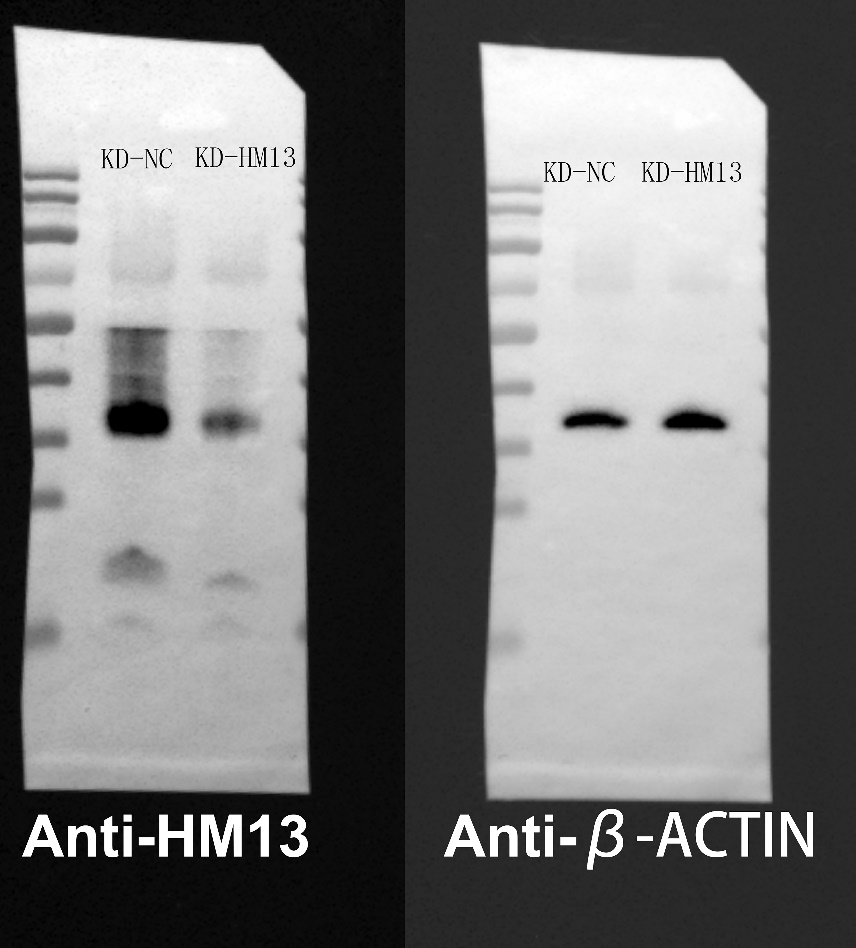

Supplement: Supplementary file 2 — Additional file 2: Fig. S1. Increased HM13 expression had poor overall survival, whether in Kaplan-Meier Plotter (A) or HPA (B) database. [file 12885_2022_9987_MOESM2_ESM.docx]
